# Synchronizing N Release from Organic Residues: Opportunities for Integrated Management of N

**DOI:** 10.1100/tsw.2001.361

**Published:** 2001-11-22

**Authors:** U. Singh, K.E. Giller, C.A. Palm, J.K. Ladha, H. Breman

**Affiliations:** Research and Development Division, International Fertilizer Development Center, Muscle Shoals, AL 35662, USA

**Keywords:** Sub-Saharan Africa, decision support tools, biological nitrogen fixation, control release N fertilizers, legumes, cereals, N release, crop N demand, organic resources, mineral fertilizers

## Abstract

In intensive cropping systems, mineral nitrogen (N) fertilizers represent the largest component of the N cycle because the indigenous N supply is not adequate. The requirement for mineral fertilizer may be reduced with the use of organic nutrient sources. A more realistic use of organic matter, particularly in sub-Saharan Africa due to limited amounts and availability, is the combined use of organic nutrient sources and mineral fertilizers. The beneficial effects of integrated use of inorganic fertilizers and organic manures on improved nutrient recoveries, soil moisture retention, cation exchange capacity, and erosion control have been reported. However, there are as many reports indicating negligible benefits or even disadvantages of combining nutrient sources on crop production. This is not surprising given the combination of organic residue sources, soils, climatic, crops, and management factors that influence nutrient dynamics. The most widely accepted function of organic materials is improving the nutrient availability to crops by supplying N. The key to both improving efficiency of N use and reducing N losses is synchronization of N supply from soil, biological N2 fixation, organic residues, and inorganic fertilizers with the crop N demand. Organic materials are not magic; N losses also result from their use. Controlling N release from organic sources depends on their nutrient content and quality, soil properties, and the environmental and management factors. This paper will synthesize the information generated from integrated nutrient management trials in sub-Saharan Africa and the Philippines. Management strategies based on an organic resources database and a dynamic soil-crop simulation model are used to identify organic sources as N fertilizers or soil amendments. The decision support tools are also used to attain optimum synchrony between release from organic sources and soils with crop N demand.

## INTRODUCTION

Intensification of agriculture requires sufficient inputs of nutrients through biological $N_2$ fixation (BNF), organic materials, and commercial fertilizers to match exports of nutrients through harvested products and losses. In many developing countries, particularly sub-Saharan Africa (SSA), the nutrient output exceeds the input resulting in widespread mining of soil nutrients and depletion of soil fertility[1,2]. Here the organic resources are in short supply and problems of poverty and poor infrastructure lead to sparse use of mineral N fertilizers. N is generally the most limiting nutrient in intensive cropping systems, and adequate N supply is required throughout the active growth period. N transformations and management in irrigated and favorable rainfed systems are well understood. In contrast, understanding process-level N dynamics in unfavorable environments, also characterized by smallholder farmers, is just beginning.

Due to high risks, farmers in unfavorable environments (climate, drought, infertile soil, poor soil moisture–holding capacities) are reluctant to apply mineral fertilizers. Decisions leading to the adoption of new technologies are very much influenced by the nature of risk and by farmers' perception of such risk. The limited and erratic rainfall and the poor nutrient-supplying capacity of the soil result in low amounts and poor-quality organic materials that supply a small unsustainable fraction of the N required by food crops. In most of the developing-country agriculture, approaches based purely on organic resources cannot offer sufficient nutrients to sustain crop yields, thereby contributing to further soil degradation. Profitable and sustainable agricultural production cannot develop under these circumstances.

Soil N and applied N (mineral fertilizers or organic materials) undergo many transformations involving numerous pathways and states, all of which are influenced by the weather, soil properties, and management practices. The need for a systems approach involving simulation models to appropriately manage N in a cropping system is evident given the complexity of soil N cycle, the myriad of pathways for N transformations, and the nuances of weather. Yet N management in most developing-country agriculture is based on trial and error. Better N management strategies that are specific for given soil types and cropping systems are urgently needed. Better prediction of optimal N rates on a field-specific and dynamic basis is a significant research and technology transfer challenge. This predictive capability would help avoid excessive N input or deficiency and also improve the profitability of N use[3]. In addition to the low N recoveries (associated with poor fertilizer recommendations), the main constraints to the use of inorganic N fertilizers in SSA are (1) limited accessibility, (2) high prices, and (3) lack of markets and price stability for excess production beyond the farmer's need.

The urgent need to combat nutrient depletion, to conserve and rejuvenate the deteriorated soil resource base, and to promote the adoption of soil fertility management techniques requires holistic, systems-based, and participatory research and resource management strategies. The rejuvenation of degraded and infertile soils undoubtedly requires inputs of nutrients; however, alternative approaches for the addition of nutrients are available. It is logical that efficient use of appropriate organic inputs supplemented with mineral fertilizers may be an optimal strategy for smallholder farmers[4]. In addition, access to better varieties, crop protection chemicals/products, and changes in management practices will be needed to achieve increased crop productivity and improved soil fertility in SSA and South Asia. This paper presents a systems-based approach to promote integrated nutrient management techniques for N to help address the declining crop production and soil fertility.

## NITROGEN SYNCHRONY

The key to both improving efficiency of applied N and reducing N losses is synchronization of N supply from soil, BNF, organic materials, and fertilizers with crop N demand[3,5]. A perfect N fertilizer would thus:

1. Provide N in the correct quantity in perfect synchrony with plant demand
2. Avoid N losses from the system, both to ensure efficient use of scarce resources and to avoid environmental problems[4]

The perfect N sources could be mineral fertilizers or organic resources. The factors influencing the synchrony are N sources; crop species; genotype; crop age; soil physical, biological, and chemical properties; and management[6].

### Plant N Demand

In annual food crops, at any point in time, there exists a critical tissue N concentration below which growth will be reduced. This concentration changes with crop growth stage. During early growth, N concentrations are usually high due to synthesis of large amounts of organic N compounds required for photosynthesis and growth. The potential N requirement for a crop is the sum of N required for new growth and the amount of N needed to overcome any existing deficiency. These components of N demand also highlight important management issues:

1. The critical N requirement differs with crop age.
2. N deficiency (diagnosed by tissue analysis, chlorophyll meter, color chart, etc.) occurs when actual N concentration falls below the critical level.

The actual rate of N uptake is dependent on crop N demand, N uptake capacity, and the N supply. The smallest of these quantities determines the actual rate of uptake for the given day.

### N Supply

The focus of this paper is whether or not we can control N supply from organic resources. First, we will consider the N supply from the soil, i.e., once N from organic materials or inorganic N fertilizers have become part of the soil (organic and inorganic) N pool. How much of the soil N from a given soil layer is used by the crop and what fraction is lost due to leaching, runoff, volatilization, and denitrification is dependent on the following:

1. Ammoniacal-N and nitrate-N concentration (inherent soil fertility, rate and timing of external N input, release rate of N from fertilizers and organic material as influenced by soil temperature, soil moisture, and soil pH)

2. Distribution of mineral N between soil solid and soil solution
3. Moisture availability (water-logging and soil oxygen)
4. Soil texture and slope (infiltration and runoff rates)
5. Root length density
6. Maximum N uptake potential per unit length of root
7. Crop N demand

The fraction that is lost from the cropping system is a source of much of the environmental pollution associated with fertilization. In addition, some of the inorganic N becomes unavailable to plants due to immobilization.

Soil N supply plays a critical role in meeting postanthesis N requirement, particularly when the last fertilizer N application is made at floral initiation (60 to 90 days before maturity) or before planting as with organic sources. Under such conditions the entire postanthesis N uptake occurred from soil N supply[7]. Hence, the timing of N application and/or N release has a crucial effect on the capability of the soil to supply the N to meet a crop's N demand.

## LEGUMES IN CROPPING SYSTEMS

The climate, soil properties, and water availability mainly determine the niches for legume growth. In rice-based cropping systems the length of the nonflooded period is also very important. Ultimately, socioeconomic and cultural practices including food habits, labor requirements, and market demand will dictate whether farmers include legumes in their cropping systems. To obtain a comparative advantage over cereals, legumes will have to benefit from conditions that enable them to exploit their BNF mechanism:

1. Flourish where soils are N deficient while available-P is relatively abundant
2. Soil contains enough Mo, Ca, and other nutrients
3. Necessary strain(s) of rhizobium are present
4. Environment with a limited pressure of pests and diseases

Soil N deficiency is such a common phenomenon that legumes could be useful almost everywhere. A severe bottleneck, however, is the low level of available-P in the soil, particularly under upland conditions in much of SSA and South Asia. Legumes are an invaluable source of protein in developing countries, but the demand for carbohydrates (energy) are higher. Thus, as long as absolute food deficiency occurs, e.g., in SSA and South Asia, the area required for producing carbohydrates should not decrease to accommodate the production of protein. Farmers are often reluctant to devote land and resources to grow legumes solely for green manure or animal feeds because they provide no immediate income or food but require labor. Hence, the dilemmas faced by farmers are how to produce enough carbohydrates and protein for their consumption and also improve soil fertility through green-manuring and N recycling. Intercropping and/or mixed cropping of food legumes or cereals with forage legumes during the dry season or the short-rains to provide food, fodder, and residue for the next wet-season crop could be attractive to farmers[8,9]. Generations of farmers in developing countries exploit the niche by "mixed cropping" – using some water and soil-P (including soil-P that may not be available to sole crop)[10] for legumes without detrimental effects to the main crop. This strategy also helps spread risks.

Legumes can partially satisfy the N requirements of the cereal crops. The theoretical maximum for high potential production environment is in the order of 50% when the complete leguminous biomass is recycled[11]. For SSA, this is achieved when legumes are grown on half of all croplands at the potential rainfall limited production – fertilized annually with 25 kg P $ha^{-1}$ and protected against pests and diseases. Where P is limiting the use of legumes is feasible only if the price of fertilizer-P is considerably less than four times the price of fertilizer-N[9]. The situation improves if the N recycling from legumes is a by-product of the legume production to feed humans or animals and where their protein requirement pays for the inputs. With food legumes, a maximum 30% of the other crop's N-requirement is met. This assumes that all leguminous biomass, with the exception of the food grains, is recycled.

In cases where the price of fertilizer-N is one quarter of the price of fertilizer-P, N-deficiency can be tackled better through N-fertilization of cereal crops instead of using P-fertilization of legumes. Using a large fraction of the land for intensive legume production is risky from the sustainability point of view because[12]:

1. In the long term soil acidification, particularly on sandy soils or soils with low pH buffering capacity, is an inevitable byproduct of legumes unless important investments are made in liming.
2. Legumes do not improve the soil organic matter status as strongly as grass-cereal rotation.

Organic matter with a much higher C/N ratio and slower mineralization rate is required to increase the soil organic matter content of West African soils (Table 1). Only then will improved water and nutrient-holding capacity and increased fertilizer use efficiency be possible[13].

## CONTROL-RELEASE N FERTILIZERS

While the price of N fertilizers may increase due to removal of subsidies and/or increased production costs, these fertilizers will remain important sources of plant N. Improving N fertilizer use efficiency both for economical and environmental reasons thus remains an important goal. One of the most convenient and economical features of inorganic N fertilizer is the option for split application so that N is available when the crop needs it. Timing of N application is one of the key N management issues. Tissue N analyses, color charts, and chlorophyll meters have been used to improve the precision of N timing. The different N-fertilizer sources are also selected based on soil types, cropping systems, and time of application.

Fertilizer products such as controlled-release N fertilizers (CRNFs) are being evaluated as sources capable of providing improved synchrony. These products have an ultrathin membrane polymer coating that encapsulates a urea granule. The controlled and predictable release of nutrients through this membrane distinguishes CRNFs from slow-release fertilizers such as sulfur- and neem-coated urea. Under a wide range of moisture

**TABLE 1**
**Average Chemical Characteristics of West African Soils (0 to 30 cm) with Decreasing Rainfall[9]**

| | pH | Org C g kg$^{-1}$ | Total N g kg$^{-1}$ | Total P g kg$^{-1}$ | P-Bray mg kg$^{-1}$ | CEC mmol kg$^{-1}$ | Base sat. % |
|---|---|---|---|---|---|---|---|
| Equatorial forest | 5.7 | 20 | 2.0 | 0.26 | 9 | 87 | 28 |
| Guinea savanna | 5.7 | 12 | 1.3 | 0.34 | 7 | 85 | 59 |
| Sudan savanna | 6.7 | 6 | 0.5 | 0.21 | 4 | 81 | 69 |
| Sahel | 5.7 | 3 | 0.2 | 0.10 | 4 | 25 | 28 |

regimes (25 to 150% field capacity) the rate of N release is independent of moisture. The rate and time of release are controlled by:

1. Membrane coating thickness
2. Ratio of high water permeable polymer to low water permeable polymer membrane
3. Temperature, with a $Q_{10}$ value of 2

Given the growth duration of a genotype and its thermal environment, it is possible to synchronize N release from CRNF with the anticipated plant N demand by selecting appropriate polymer membrane coating and thickness. Fertilizer recovery with CRNF has been 70 to 75%, compared with 35 to 50% with prilled urea[3]. CRNF also provides the opportunity for placement of fertilizer with seed – co-situs placement. The co-situs placement has the following advantages:

1. Reduced labor cost (single operation for seeding and N fertilization)
2. Better contact between fertilizer and root
3. Increased fertilizer efficiency
4. Reduced N losses (leaching, gaseous emission, runoff)
5. Reduced weed competition (less fertilizer N for weeds)
6. Microbial activity is not inhibited by high concentration of N in the soil

## N RELEASE FROM ORGANIC RESIDUES

Unlike mineral fertilizers, organic residues must decompose before their N becomes available to the plant in mineral form. Achieving synchronization of N mineralization from applied organic fertilizers and N demand for a given target yield level may reduce N losses and increase N use efficiency [14]. The factors that determine the dynamics of residue breakdown include: cultural practices, soil properties (including microfauna, organic matter content, pH, temperature, and soil moisture), and chemical composition of organic material. Cultural practices under lowland conditions include duration of submergence, method of residue incorporation, and application of mineral N[15]. Chemical compositions of organic residues influencing N release are carbon (C), N, lignin (L), and polyphenol ($P_p$) content, and their ratios such as C-to-N ratio, L-to-N ratio, $P_p$-to-N ratio, and L+$P_p$-to-N ratio.

### Upland Soils

The $P_p$-to-N and the L+$P_p$-to-N ratios are probably the most reliable parameters to predict residue N mineralization in upland soils[16,17]. Synthesis of research on plant residue quality and decomposition on upland soils has led to the development of an Organic Resource Database[18]. Based on the database a decision tree to guide use of organic resources for soil amendment was developed (Fig. 1). The essence of the decision tree and the chemical quality controls is that they are hierarchical in nature, e.g., IF N > 2.5%; only then polyphenols are important, otherwise they are not. As previously noted, approaches solely based on organic residues and BNF are unlikely to satisfy the production demands of N in most regions. The potential for enhanced benefits from the combined use of organic resources and mineral N fertilizers needs to be quantified and promoted.

### Lowland Condition

N mineralization was not significantly correlated with N content of the residues, C-to-N ratio, moisture content, or polyphenol contents under lowland conditions[19]. In contrast to tropical upland soils[17], polyphenols showed no apparent interaction with N mineralization in flooded soils and combining soluble polyphenols with nonsoluble lignin in L-to-N ratio (L+$P_p$-to-N ratio) did not improve correlation coefficients. The dilution of these water-soluble compounds in soil solution and floodwater rice soils apparently limits their reaction with N compounds[20]. For residues low in polyphenols, the L-to-N ratio seems to suitably predict their N mineralization rate in flooded soils, but this relation varies from soil to soil. Initial net $NH_4^+$-N mineralization rates were higher from residues with relatively low L-to-N ratio (*Sesbania rostrata*, L-to-N ratio 2) than from material with higher L-to-N ratio (*S. emerus*, L-to-N ratio 6, and *S. rostrata*-rice straw mixture, L-to-N ratio 6). Rice straw alone (L-to-N ratio 12) resulted in an initial net N immobilization. Manipulating the L-to-N ratio of an organic material by mixing high-quality material (e.g., legumes) with crop residues (e.g. straw) that are available in rice fields is a simple and effective way to control residue N mineralization in flooded soils[21].

## DECISION SUPPORT SYSTEM (DSS)

Synchrony of N release with crop N demand will not increase yields if amount of N available is limiting; however, it will re-

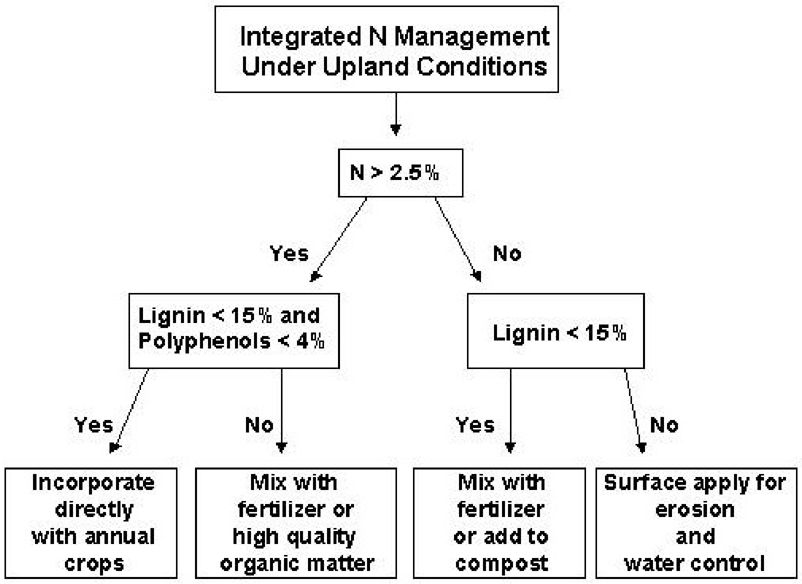

**FIGURE 1**. A decision tree on the use of organic resources for integrated nutrient management[18].

duce N losses and improve long-term soil fertility. In situations where a short-term yield reduction is the price for long-term benefits, the application of N supply-demand synchrony based solely on organic resources may not be attractive to farmers. Decision support tools are needed to optimize synchrony for a range of soils, organic residues, and timing of mineral N fertilizers. Nutrient management should also target the needs of crop rotations rather than focusing on individual crops because application of nutrients such as P can have strong residual benefit over several crop cycles[4]. Decision support tools should provide flexible guidelines for management of both organic and mineral nutrient resources rather than the fixed fertilizer recommendations that remain the rule in most developing countries, particularly in the SSA.

Decision Support System for Agrotechnology Transfer (DSSAT)[22], linked with the Organic Resource Database[18] was used to predict not only N supply but also the N release pattern and its synchrony with plant N demand. The effect of four organic residues – maize stover (0.6% N, 8% lignin, 1% polyphenols), mucuna tops (3.0% N, 8% lignin, 1.8% polyphenols), pigeon pea stover (3% N, 18% lignin, 1.5% polyphenols), and leucena leaves (3% N, 11% lignin, 5.2% polyphenols) as the N source, each applied at 2 t ha$^{-1}$ was evaluated using the modified DSSAT model at Zaria, Nigeria. The results (Fig. 2)

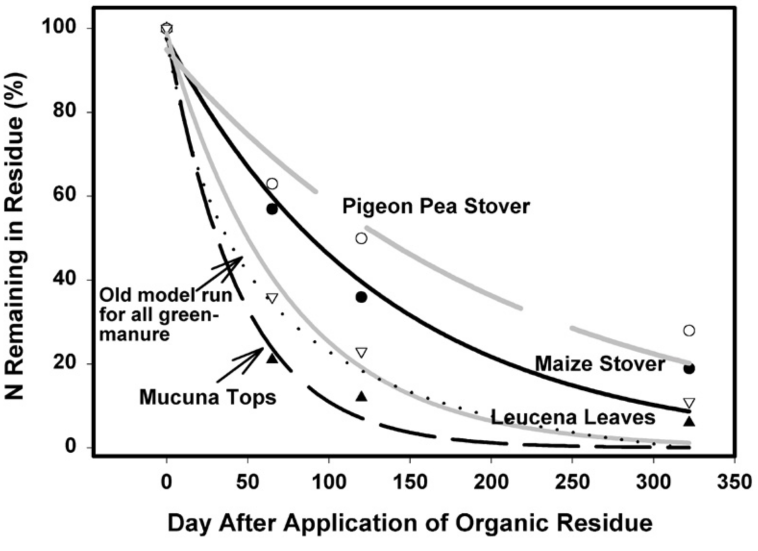

**FIGURE 2**. Observed (symbols) and simulated (lines) percentage of N remaining in four different organic residues after field incubation with Zaria soil. Dotted line represents results from the previous version of the model with mucuna, pigeon pea, and leucena.

illustrate the model's improved capability to simulate N mineralization for the four organic sources. The previous version of the model was not able to simulate different N releases from the three green manure residues (mucuna, pigeon pea, and leucena) with similar N (3% N) but varying lignin and polyphenolic content. Based on the amount and the timing of N release, appropriate organic N sources could be selected. For mucuna tops, with high N concentration and fast N release, the timing of residue application must synchronize with plant N demand to avoid excessive N losses.

These simulations were limited to the N-supplying capacity of the residues. In the infertile soils of SSA, organic matter additions would also play a critical role in improving water retention, reducing soil erosion, and improving nutrient recovery through their impact on CEC, root penetration, and soil organisms. The above simulations also assumed that organic residues once applied stayed in the field. In SSA, anything from termites to cattle could consume and thus remove the organic residue from the target fields to elsewhere. It is thus very important for decision makers to consider abiotic, biotic, and sociocultural factors when making recommendations for yield and soil fertility improvement. It is equally important to realize the limitations and the usefulness of the existing DSS.

## CONCLUSIONS

The multitude of soil, climate, and management factors together and the types of organic sources influence N mineralization and N supply to crops. An organic resources database combined with a computer simulation model was used to simulate N release and optimize N synchrony. The simulations indicated that some organic resources basally applied followed by a top-dressing with mineral N resulted in improved synchrony with crop N demand. The integrated use of organic and inorganic N sources also helps agricultural intensification, improved productivity, and environmental protection. The DSS has not been optimized in terms of development and validation at present; hence, it is important to realize the limitations.

## ACKNOWLEDGMENTS

We are grateful to the system-wide Soil Water and Nutrient Management Program of the CGIAR for their partial support of the work reported. Appreciation is extended to Drs. Richard Ogoshi and Deborah Hellums for their helpful comments.

| | | |
|---|---|---|
| Received: | July | 31, 2001 |
| Revised: | November | 5, 2001 |
| Accepted: | November | 7, 2001 |
| Published: | November | 22, 2001 |
